# Draft genome sequence of *Lactiplantibacillus plantarum* LAB20 isolated from the gut of silver therapon (*Leiopotherapon plumbeus*), a probiotic candidate with anti-*Vibrio parahaemolyticus* activity

**DOI:** 10.1128/mra.00569-26

**Published:** 2026-07-16

**Authors:** Nico G. Dumandan, Annie Cita T. Kagaoan, Eldrin D. L. R. Arguelles

**Affiliations:** 1 National Institute of Molecular Biology and Biotechnology (BIOTECH), University of the Philippines Los Baños 54729, Laguna, Philippines; Fluxus Inc., Sunnyvale, California, USA

**Keywords:** *Lactiplantibacillus plantarum*, gut microbiome, probiotic candidate, aquaculture, *Vibrio parahaemolyticus*

## Abstract

Gut-associated lactic acid bacteria are promising candidates for aquaculture probiotics. Here, we present the draft genome of *Lactiplantibacillus plantarum* LAB20 from silver therapon (*Leiopotherapon plumbeus*). Genomic features supporting adhesion, stress tolerance, and antimicrobial activity against *Vibrio parahaemolyticus* were identified, although further studies are needed to evaluate safety.

## ANNOUNCEMENT

The fish gastrointestinal microbiome is a valuable source of microorganisms with potential applications in aquaculture. In this study, strain LAB20 was isolated from the gut of silver therapon (*Leiopotherapon plumbeus*), an endemic freshwater fish in the Philippines. Wild-caught juveniles obtained from the Bureau of Fisheries and Aquatic Resources Regional Office No. 5 (Fabrica, Bula, Camarines Sur, Philippines; 13.49836°N, 123.30441°E) were dissected aseptically, and excised gut samples were homogenized in sterile phosphate-buffered saline, serially diluted, and plated on de Man, Rogosa, and Sharpe (MRS) agar supplemented with 1% calcium carbonate. Plates were incubated at 37°C for 48 h in a candle jar. Colonies exhibiting calcium carbonate dissolution halos were purified by repeated streaking on MRS agar and screened for antagonistic activity against *Vibrio parahaemolyticus* by agar well diffusion assay as previously described (1). For preliminary taxonomic identification, strain LAB20 was cultured in MRS broth at 37°C for 24 h under static conditions in screw-capped tubes. Genomic DNA was extracted using InstaGene Matrix (Bio-Rad Laboratories, USA), and the nearly full-length 16S rRNA gene was amplified using primers 27F (5′-AGAGTTTGATCMTGGCTCAG-3′) and 1492R (5′-TACGGYTACCTTGTTACGACTT-3′). Amplicons were bidirectionally Sanger sequenced on an ABI PRISM 3730XL DNA Analyzer (Applied Biosystems, Foster City, CA, USA). BLASTn analysis of the 16S rRNA gene sequence showed the highest similarity to *Lactiplantibacillus plantarum* JCM 1149 (GenBank accession no. NR_115605.1) and high similarity to *Lactiplantibacillus pentosus* 124-2 (GenBank accession no. NR_029133.1). Given their high sequence similarity, whole-genome sequencing was performed for definitive species-level identification.

For whole-genome sequencing, strain LAB20 was independently cultured under same conditions. Genomic DNA was extracted using the NucleoSpin Microbial DNA Isolation Kit (Macherey-Nagel, Germany), assessed and quantified using a BioSpec-nano spectrophotometer (Shimadzu Corp., Kyoto, Japan), and sequenced by Macrogen, Inc. (Seoul, South Korea). A TruSeq Nano DNA Library with a 350-bp insert size was sequenced on an Illumina HiSeq platform, generating 24,687,044 paired-end reads (150 bp). Raw reads were assessed using FastQC v0.12.1 (2), and no trimming was required because of high read quality.

Genome assembly was performed using SPAdes v4.2.0 (3), followed by iterative polishing with Pilon v1.24 (4). Annotation was performed using the National Center for Biotechnology Information (NCBI) Prokaryotic Genome Annotation Pipeline (PGAP) revision 6.11 employing the Best-Placed Reference Protein Set and GeneMarkS-2+ methods (5). Functional classification was performed using eggNOG-mapper v5.0.2 (6), and genome quality was assessed using CheckM v1.2.5 (7), which estimated 98.91% completeness and 3.03% contamination. Secondary metabolite biosynthetic gene clusters were identified using antiSMASH v8.0 (8). All kits were used according to the manufacturers’ instructions, and all software analyses were performed using default parameters unless otherwise specified. Key genome assembly, quality, and annotation features are summarized in Table 1.

**TABLE 1:** Genome assembly, quality, and annotation features of strain LAB20

Genome features	Value
Assembly statistics^*a*^	
Genome size (bp)	3,259,645
Total ungapped length (Mb)	3.3
Number of contigs	320
Contig N50	23.1 kb
Contig L50	40
GC content (%)	44.5
Genome coverage	50×
Genome quality^*b*^	
Genome completeness (%)	98.91
Genome contamination (%)	3.03
Annotation features^*a*^	
Total number of genes	3,239
Total number of CDS	3,157
Total number of coding CDS	3,140
Total number of RNA genes	82
Pseudogenes	17
rRNAs	1,3,5 (5S, 16S, and 23S)
tRNAs	69
ncRNAs	4

^*a*^ Derived from PGAP rev. 6.11 genome annotation based on the Best-Placed Reference Protein Set and GeneMarkS-2+ gene prediction pipeline.

^*b*^ Derived from CheckM v1.2.5.

Species identity was confirmed by average nucleotide identity (ANI) analysis using FastANI v1.34 (9). The assembled genome shared 99.06% ANI with *Lactiplantibacillus plantarum* (GenBank accession no. CP058967.1) and 82.49% ANI with *Lactiplantibacillus pentosus* (GenBank accession no. CP129655.1), confirming LAB20 as *L. plantarum*. Functional annotation identified genes associated with stress adaptation, host colonization, and antimicrobial activity. antiSMASH analysis identified terpene, type III polyketide synthase (T3PKS), cyclic lactone-associated, and RiPP-like clusters.

## Data Availability

The whole-genome shotgun project has been deposited in GenBank under accession number JBXGSX000000000. The BioProject accession PRJNA1455347 includes the BioSample (SAMN57318923) and SRA (SRR38146721) records associated with this study.

## References

[1] Dumandan NG, Kagaoan ACT, Labitag LJF, Arreola SLB. 2024. Fermentability assessment of selected oligosaccharides by *Lactiplantibacillus plantarum* BIOTECH 1074 towards short-chain organic acid production with anti-*Vibrio* efficacy. Bioresource Technology Reports 27:101922. doi:10.1016/j.biteb.2024.101922

[2] Andrews S. 2010. FastQC: a quality control tool for high throughput sequence data. Cambridge, United Kingdom

[3] Bankevich A, Nurk S, Antipov D, Gurevich AA, Dvorkin M, Kulikov AS, Lesin VM, Nikolenko SI, Pham S, Prjibelski AD, et al.. 2012. SPAdes: a new genome assembly algorithm and its applications to single-cell sequencing. J Comput Biol 19:455–477. doi:10.1089/cmb.2012.0021 22506599 PMC3342519

[4] Walker BJ, Abeel T, Shea T, Priest M, Abouelliel A, Sakthikumar S, Cuomo CA, Zeng Q, Wortman J, Young SK, et al.. 2014. Pilon: an integrated tool for comprehensive microbial variant detection and genome assembly improvement. PLoS One 9:e112963. doi:10.1371/journal.pone.0112963 25409509 PMC4237348

[5] Tatusova T, DiCuccio M, Badretdin A, Chetvernin V, Nawrocki EP, Zaslavsky L, Lomsadze A, Pruitt KD, Borodovsky M, Ostell J. 2016. NCBI prokaryotic genome annotation pipeline. Nucleic Acids Res 44:6614–6624. doi:10.1093/nar/gkw569 27342282 PMC5001611

[6] Huerta-Cepas J, Forslund K, Coelho LP, Szklarczyk D, Jensen LJ, von Mering C, Bork P. 2017. Fast genome-wide functional annotation through orthology assignment by eggNOG-mapper. Mol Biol Evol 34:2115–2122. doi:10.1093/molbev/msx148 28460117 PMC5850834

[7] Parks DH, Imelfort M, Skennerton CT, Hugenholtz P, Tyson GW. 2015. CheckM: assessing the quality of microbial genomes recovered from isolates, single cells, and metagenomes. Genome Res 25:1043–1055. doi:10.1101/gr.186072.114 25977477 PMC4484387

[8] Blin K, Shaw S, Vader L, Szenei J, Reitz ZL, Augustijn HE, Cediel-Becerra JDD, de Crécy-Lagard V, Koetsier RA, Williams SE, et al.. 2025. antiSMASH 8.0: extended gene cluster detection capabilities and analyses of chemistry, enzymology, and regulation. Nucleic Acids Res 53:W32–W38. doi:10.1093/nar/gkaf334 40276974 PMC12230676

[9] Jain C, Rodriguez-R LM, Phillippy AM, Konstantinidis KT, Aluru S. 2018. High throughput ANI analysis of 90K prokaryotic genomes reveals clear species boundaries. Nat Commun 9:5114. doi:10.1038/s41467-018-07641-9 30504855 PMC6269478

